# Investigation of Electrical Discharge Processes During Electrolytic–Plasma Nitrocarburizing

**DOI:** 10.3390/ma18143381

**Published:** 2025-07-18

**Authors:** Bauyrzhan Rakhadilov, Laila Sulyubayeva, Almasbek Maulit, Temirlan Alimbekuly

**Affiliations:** 1 PlasmaScience LLP, Oskemen 070018, Kazakhstan; rakhadilovb@mail.ru; 2 Research Center Surface Engineering and Tribology, Sarsen Amanzholov East Kazakhstan University, Oskemen 070000, Kazakhstan; lsulyubayeva@gmail.com (L.S.); temirlanalimbekuly@gmail.com (T.A.); 3 Research School of Physical and Chemical Sciences, Shakarim University, Semey 071412, Kazakhstan

**Keywords:** electrolytic–plasma nitrocarburizing, spectral analysis, high-speed imaging, microstructure, friction coefficient, temperature regime

## Abstract

In this study, the process of electrolytic–plasma nitrocarburizing (EPNC) of 20-grade steel was investigated using various electrolytes and temperature regimes. At the first stage, optical spectral analysis of plasma emission during EPNC was carried out with spectral registration in the range of 275–850 nm, which allowed the identification of active components (Hα, CN, Fe I, O I lines, etc.) and the calculation of electron density. Additionally, the EPNC process was recorded using a high-speed camera (1500 frames per second), which made it possible to visually evaluate the dynamics of arc and glow discharges under varying electrolyte compositions. At the next stage, the influence of temperature regimes (650 °C, 750 °C, and 850 °C) on the formation of the hardened layer was studied. Using SEM and EDS methods, the morphology, phase zones, and the distribution of chemical elements were determined. Microhardness measurements along the depth and friction tests were carried out. It was found that a temperature of 750 °C provides the best balance between the uniformity of chemical composition, high microhardness (~800 HV), and a minimal coefficient of friction (~0.48). The obtained results confirm the potential of the selected EPNC regime for improving the performance characteristics of 20-grade steel.

## 1. Introduction

Enhancing the wear resistance and service life of metal components is often achieved through thermochemical surface treatments, such as nitriding or carburizing. A hardened surface with high hardness and wear resistance significantly extends the service life of components such as axles, shafts, pins, gears, bushings, and other mechanical elements. To enhance the service life and reliability of heavily loaded components, it is necessary to form a property gradient: a hard and wear-resistant surface layer while maintaining a tough and impact-resistant core. In engineering practice, various thermal and thermochemical treatment methods are used to achieve this, such as bulk hardening followed by tempering, carburizing, nitrocarburizing, gas and ion nitriding, as well as local surface strengthening techniques based on high-frequency current (induction hardening) or concentrated energy fluxes (laser hardening) [1,2,3]. These approaches have proven effective in improving the hardness and fatigue strength of steels, significantly extending the service life of components such as gears and shafts [4,5]. However, alongside their advantages, traditional methods also have a number of limitations. For example, bulk hardening requires heating the entire component followed by cooling, which may lead to significant thermal and structural distortions. Local methods, such as induction and laser hardening, allow for strengthening of specific areas, but they require expensive equipment and are associated with high energy consumption [6,7]. Traditional processes (such as gas nitriding, furnace-based nitrocarburizing, etc.) require prolonged exposure at elevated temperatures and involve the use of toxic media (e.g., ammonia, cyanates), which drives the search for more efficient and environmentally friendly alternatives [8,9,10]. In recent decades, the technology of plasma–electrolytic treatment (PET) has been developed, which enables the surface modification of metals in aqueous electrolytes under the influence of a gas envelope and plasma discharges [11]. Electrolytic–plasma nitrocarburizing (EPNC) is of particular interest—it is a diffusion-based process of enriching the steel surface with nitrogen and carbon under plasma conditions formed in an aqueous solution at high voltage [12,13]. This process combines nitriding and carburizing (nitrocarburizing) with rapid thermal surface treatment (nitroquenching), enabling the formation of a hard, wear-resistant layer within just a few minutes [7,14].

During electrolytic–plasma treatment, a stable vapor–gas envelope forms around the heated metal electrode due to the boiling of the adjacent solution layer [13,14]. After reaching the threshold voltage (~250–400 V in aqueous electrolytes), the envelope transitions into a mode of plasma micro-arc discharges [15]. In the cathodic mode, these discharges concentrate near the surface of the workpiece (cathode), locally generating high-temperature plasma (temperature > 800–1000 °C) [16]. Around each micro-arc, a microplasma cloud is formed—an ionized gas containing electrons, ions, excited neutral atoms, and radicals. The ensemble of microdischarges forms a luminous plasma envelope over the entire surface of the electrode [17]. Discharges in liquid media are classified into three main groups: discharges occurring directly within the liquid volume [18]; discharges taking place in the gas phase above the liquid surface, including cases where the liquid itself serves as a conductive electrode [19]; and discharges in multiphase systems, such as bubbles, vapor–gas mixtures, aerosols, and foams [20]. To study the plasma formed under such conditions, visual observation and optical emission spectroscopy are commonly used [21]. These methods provide important information about the nature of plasma formations, their geometry, composition, temperature, and the density of constituent components [22,23]. Aqueous solutions containing nitrogen and carbon compounds are used for conducting EPNC. Most commonly, urea CO(NH_2_)_2_, ammonium salts (such as NH_4_Cl, NH_4_NO_3_), and organic additives (e.g., glycerol, formamide, etc.) are employed—they serve as sources of both nitrogen and carbon [14,17]. The composition of the electrolyte significantly affects the characteristics of the plasma. Studies have shown that a higher water concentration leads to the formation of a thicker Fe_3_O_4_ oxide film on the surface, which slightly reduces the corrosion resistance of the layer [24]. In contrast, the use of urea or ammonium nitrate with a moderate water content leads to the formation of a predominantly nitrocarburized diffusion layer with minimal porosity [25,26]. The duration of the EPN process is generally short—ranging from a few seconds to several tens of minutes. It has been established that the layer grows rapidly during the initial minutes and then slows down. For example, within 5 min, a layer approximately 50 μm thick can be formed, while an additional 5 min results in only about 20–30 μm more [27].

Intense electrical discharge exposure in the solution leads to the formation of a complex structural-phase state in the near-surface layer of low-carbon steel. After electrolytic–plasma nitrocarburizing, a diffusion layer of variable thickness (typically 50–300 μm) enriched with nitrogen, carbon, and accompanying elements is observed in the steel. The phase composition of such a layer typically includes iron nitrides ε-Fe_2–3_N (epsilon phase) and γ′-Fe_4_N, iron carbides of the cementite type Fe_3_C, and iron carbonitrides (carbo-nitrides) of mixed composition Fe_2–3_(N, C) [17,28]. In some cases, γ′-Fe_4_N nitrides are also detected (especially with an increased content of alloying elements that stabilize this phase), along with complex compounds such as iron ferrocyanides, if the corresponding components were present in the electrolyte [29,30]. Electrolytic–plasma nitrocarburizing provides a significant increase in the surface hardness of steels. Due to the formation of iron nitrides (with a hardness of ~800–1100 HV) and martensitic hardening of the diffusion zone, the microhardness of the outer layer in low-carbon steels reaches 700–1000 HV, whereas the base steel has a hardness of ~150–200 HV. The hardness gradually decreases through the layer thickness: in the diffusion zone, a gradient is observed—from ~600 HV just below the compound zone to ~300 HV at a depth of ~0.2 mm [31]. For example, after EPNC of Q235 steel in an ethanolamine solution, the surface hardness reached ~750 HV, which is 4.5 times higher than that of the untreated material [29]. Similarly, during nitrocarburizing of AISI 1045 steel in urea, the microhardness reached up to 1250 HV [28]. Studies have shown that anodic EPNC increases the wear resistance of medium-carbon steel by approximately 40% compared to conventionally quenched steel under the same friction conditions [32]. This effect is explained by the presence of a hard ε/γ′ nitride phase (with high microhardness of ~8–10 GPa) and a martensitic sublayer that hinders crack initiation. The wear mechanism of the nitrocarburized layer is predominantly fatigue-related, involving micro-fracturing of nitride inclusions; however, due to their fine dispersion, wear progresses slowly [33].

In this study, the influence of electrolyte composition and the temperature regime of electrolytic–plasma nitrocarburizing on the characteristics of electrical discharge processes, as well as on the structure, microhardness, and tribological properties of 20-grade steel, was investigated with the aim of optimizing the treatment conditions.

## 2. Materials and Methods

Low-carbon steel 20, which is an analogue of AISI 1020 steel, was selected for conducting the EPNC process. The chemical composition of steel 20 according to GOST 1050-88 [34] is as follows: carbon (C) 0.17–0.24%, silicon (Si) 0.17–0.37%, chromium (Cr) up to 0.25%, manganese (Mn) 0.35–0.65%, nickel (Ni) up to 0.3%, copper (Cu) up to 0.25%, phosphorus (P) up to 0.035%, sulfur (S) up to 0.04%, and arsenic (As) up to 0.08%. The samples were prepared in the form of cubes with dimensions of 20 × 20 × 20 mm. Before the process, the sample surfaces were prepared using a HYMP-2 grinding and polishing machine (Shanghai Ju Hui Instrument Manufacturing Co., Ltd., Shanghai, China), achieving a surface roughness level of Ra 1.0 ± 0.1 μm. After polishing, the samples were thoroughly rinsed with running water to remove residual abrasive materials and any contaminants. The treatment parameters and the electrolyte composition used are presented in Table 1.

**Figure 1:**
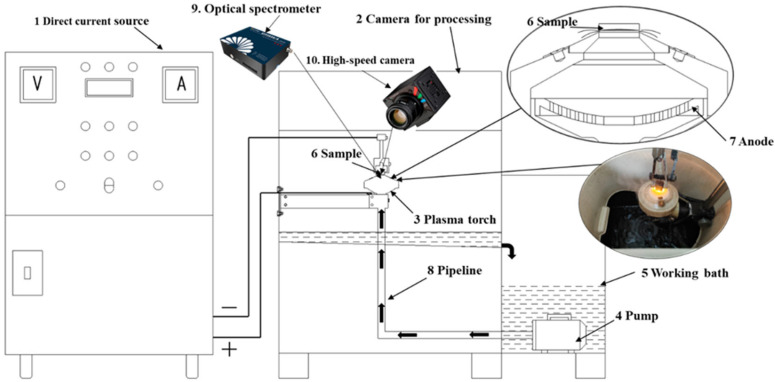
Setup for chemical-thermal treatment of materials.

The experiment was carried out using a setup for chemical-thermal treatment, equipped with a 40 kW DC power supply and a specialized treatment chamber, which housed the plasmatron, as shown in Figure 1.

The electrolytic–plasma nitrocarburizing of the samples was performed under the following procedure. Initially, the working bath (5) was filled with electrolyte, which was circulated using a pump (4) located at the base of the system. The electrolyte was pumped through pipelines (8) into the plasmatron (3), positioned inside the treatment chamber (2). After interacting with the plasmatron, the liquid returned to the bath via drainage openings, ensuring a continuous circulation loop. The electrolyte flow was regulated within 4–7 L/min. To maintain the electrolyte temperature within the 40–50 °C range, a heat exchanger was utilized, with cooling water supplied at a flow rate of 3–6 L/min. The sample (6) was mounted in a holder such that its surface faced the nozzle of a conical partition at a distance of 2–3 mm. This configuration allowed the electrolyte jet to impinge directly onto the surface, providing both cooling and temperature stabilization. For the electrical setup, the anode (7) was connected to the positive output of the power source (1), while the sample served as the cathode, connected to the negative terminal. Surface activation and rapid heating to the required saturation temperature were achieved by applying an initial voltage of 320 V for 10 s. Subsequently, the voltage was reduced to 180 V, and the treatment was sustained for 5, 7, or 10 min, depending on the selected regime. The sample temperature during the process was maintained by regulating the electrolyte flow rate [35]. During the treatment process, the discharge was recorded using a high-speed camera and an optical spectrometer.

The spectral characteristics of the electrolytic–plasma nitrocarburizing process were examined using a high-resolution optical spectrometer ATP3030 (Optosky, Xiamen, China). To identify the emission lines, the recorded spectra were compared with reference data from the National Institute of Standards and Technology (NIST) database [36]. The visual characteristics of the electrolytic–plasma nitrocarburizing process were studied using a high-speed camera Evercam HR 2000-16-C (General Optics, Moscow, Russia). X-ray phase analysis was performed using an X’Pert PRO PANalytical diffractometer (PANalytical BV, Almelo, The Netherlands) with a copper anode tube operated at 40 kV and 30 mA. Cu-Kα radiation (λ = 1.541 Å) was recorded in the 2 θ range from 30° to 100°, with a step size of 0.02° and a counting time of 0.5 s per step. The processing and interpretation of the diffraction patterns were carried out using HighScore Plus software (version 3.0e). The microstructure and energy-dispersive analysis of the nitrocarburized samples were examined using a scanning electron microscope SEM3200 (CIQTEK Co., Ltd., Hefei, China) after etching in a 4% nitric acid solution. Hardness was measured using a FISCHERSCOPE HM2000 microhardness tester under a load of 100 mN (Helmut Fischer GmbH, Sindelfingen, Germany). The coefficient of friction was determined using a universal tribological tester TRB3 (Anton Paar, Graz, Austria) according to the ball-on-disk method in compliance with ASTM G99 standard [37]. Tribological tests were performed under dry sliding conditions at a controlled room temperature of 25 ± 1 °C, with a sliding velocity of 0.05 m/s. The samples were tested against a 100Cr6 steel counterbody under a normal load of 10 N. Friction coefficient values were measured after the specimens had covered a total sliding distance of 60 m.

## 3. Results and Discussion

**Figure 2:**
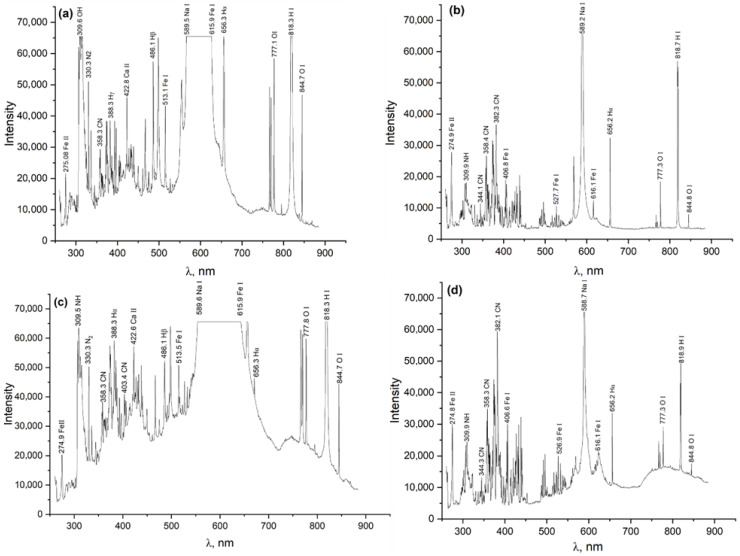
Spectrum of the electrical discharge during electrolytic–plasma nitrocarburizing in various electrolytes. (**a**) Electrolyte 1 in the beginning of the process; (**b**) Electrolyte 1 after stabilization; (**c**) Electrolyte 2 in the beginning of the process; (**d**) Electrolyte 2 after stabilization.

In Figure 2a–d, the spectrograms display intense emission lines characteristic of electrolyte dissociation products, steel components, and excited atomic and molecular species formed in the zone of the electrolytic–plasma discharge. The key identified lines observed in all spectra include hydrogen Balmer series lines—Hα (656.3 nm), Hβ (486.1 nm), and Hγ (388.3 nm), indicating a high hydrogen content in the plasma originating from water, urea, and glycerol; CN radicals (358–382 nm), which are decomposition products of urea and glycerol, and are intensified in the presence of organic compounds; NH and N_2_ (309–330 nm), representing dissociation products of ammonium chloride and urea; iron lines (Fe I, Fe II) in the range of 275–620 nm, indicating evaporation and ionization of the metallic surface of 20-grade steel; Na I lines (589.0–589.6 nm), which are traces of sodium, likely originating from sodium carbonate (soda ash); and O I lines (777–845 nm), representing atomic oxygen, an active participant in redox reactions at the steel/electrolyte interface. In spectra Figure 2a,b obtained without glycerol, more intense Fe I and Hα lines are observed, indicating higher temperature and current density in the plasma zone. In spectra Figure 2c,d, where 3% glycerol was additionally introduced into the electrolyte, the CN and OH bands are intensified, and broadened regions appear in the 770–850 nm range (OI, HI). This is attributed to the pyrolysis of glycerol and the activation of carbon–oxygen radicals. The width of the Hα line (656.3 nm) serves as a key parameter for determining the electron density of the plasma. The greatest line width, and thus the highest electron density, is observed in spectra Figure 2a,b, while the lowest is observed in spectrum Figure 2d. A comparison of the four spectra allows the following conclusions to be drawn: electrolytic–plasma nitrocarburizing is accompanied by the formation of intense hydrogen, oxygen, and iron plasma; the addition of glycerol to the electrolyte composition leads to a noticeable change in the spectral profile—specifically, an enhancement of the CN and O I lines, which confirms its active involvement in discharge and diffusion processes.

Considering the specifications of the experimental setup, the spectral line broadening due to pressure and the linear Stark effect was estimated by determining the full width at half maximum (FWHM) of the Lorentzian line shape, ∆λL, using the following formula [38]:

$${∆\lambda }_{F}=0.5346\times {∆\lambda }_{L}+\sqrt{0.2166\times {∆\lambda }_{L}^{2}+{∆\lambda }_{G}^{2}}$$

In this context, ∆λG denotes the full width at half maximum (FWHM) of the Gaussian component, representing the instrumental broadening of the Optosky ATP3030 spectrometer (equal to 1.0 nm); ∆λF corresponds to the FWHM of the resulting Voigt profile, while ∆λL refers to the Lorentzian FWHM. Based on the calculations, the Lorentzian profile’s FWHM was determined to be 0.496 nm.

The electron density (n_e_) was estimated using an empirical relation that connects the full width at half maximum (FWHM) of the Hα spectral line to n_e_ [39]:

$$n_{e}=10^{13}{\left({∆\lambda }_{L}\right)}^{\frac{3}{2}}\left[C_{0}\left(T\right)+\sum_{n=1}^{m}C_{n}(T){\left(\ln{∆\lambda }_{L}\right)}^{n}\right]$$

where ∆λL is the Lorentzian line width, and C_n_(T) is taken from [25] for the corresponding Balmer series hydrogen line. The calculated electron density, full width at half maximum (FWHM) of the Voigt profile, and FWHM of the Lorentzian profile are presented in Table 2.

**Figure 3:**
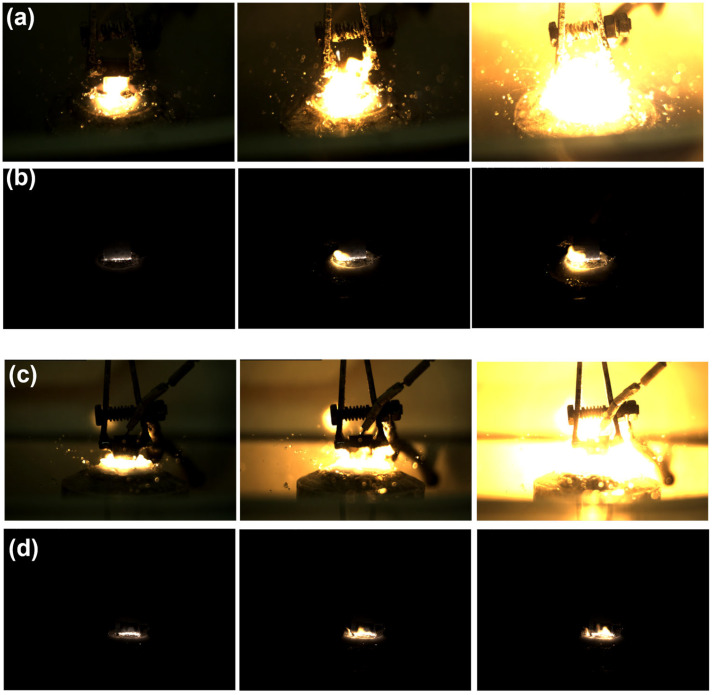
Frames of electrical discharge during electrolytic–plasma nitrocarburizing in various electrolytes. (**a**) Electrolyte 1 in the beginning of the process; (**b**) Electrolyte 1 after stabilization; (**c**) Electrolyte 1 in the beginning of the process; (**d**) Electrolyte 2 after stabilization.

The presented frames, obtained using a high-speed camera Evercam HR 2000-16-C (General Optics, Moscow, Russia) at a recording rate of 1500 frames per second, visualize the characteristic stages of the electrolytic–plasma nitrocarburizing process of 20-grade steel at a temperature of 750 °C in various aqueous electrolyte compositions. Each sequence of images (Figure 3a–d) corresponds to a specific stage of the discharge (onset of the arc or stabilized glow mode) and is consistent with the previously discussed spectroscopic data. The frames capture an intense arc discharge occurring within the first 10 s after applying a voltage of 320 V in the electrolyte containing 10% soda ash, 15% urea, and 5% ammonium chloride (Figure 3a). A bright plasma zone is visible with numerous microbursts and liquid splashing, indicating intense electrical discharge and thermo-mass transfer processes. This regime is accompanied by the appearance of prominent Fe I, Fe II, Hα, CN, and NH lines in the spectrum, indicating high temperature and active ionization of the electrolyte and steel. Figure 3b captures the glow discharge observed after plasma stabilization upon reducing the voltage to the operating level (typically 180 V). The plasma glow is uniform and localized, with reduced brightness, characteristic of the microplasma regime. The corresponding spectrum shows a narrowing of the Hα line, indicating a decrease in electron density to approximately 1.6 × 10^16^ cm^−3^, while active CN, Fe I, Na I, and O I lines remain present. Such a phase is preferable for the formation of a uniform diffusion layer on the steel surface. Figure 3c is similar to Figure 3a, but the process is conducted in an electrolyte with the addition of 3% glycerol, which visually results in enhanced light emission, increased turbulence, and more pronounced glow near the sample surface. Glycerol, as an organic component, enriches the plasma with carbon- and hydrogen-containing radicals, as confirmed by the more intense spectral lines of CN, OH, Hα, and O I. Figure 3d shows a stabilized glow discharge in the electrolyte containing glycerol. The images display a compact, stable glow corresponding to a uniform microplasma current. In spectrogram Figure 2d, a reduction in the width of the Hα line to ~0.26 nm is observed, which corresponds to a decrease in electron density to approximately 1.0 × 10^16^ cm^−3^. The presence of glycerol contributes to an increase in the intensity of CN and O I lines, which may enhance surface activity and the thermochemical saturation of the steel with nitrogen and carbon.

**Figure 4:**
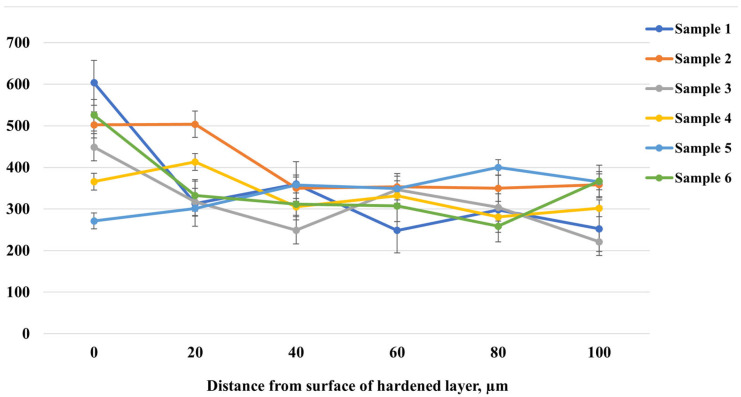
Microhardness distribution with depth after EPNC in various electrolytes.

Figure 4 shows the microhardness distribution as a function of depth from the surface of the hardened layer after EPNC of 20-grade steel under different electrolyte compositions and processing conditions. Samples 1–3 were treated in an electrolyte containing 10% soda ash, 15% urea, 5% ammonium chloride, and the remainder distilled water. The treatment was carried out at a voltage of 320 V for 10 s (to initiate the arc discharge), followed by 180 V for 5, 7, and 10 min, respectively. Samples 4–6 were treated in a similar electrolyte with the addition of 3% glycerol, under the same time regimes. Analysis of the curves shows that sample 2, treated for 7 min, exhibits the most uniform microhardness distribution with depth. Unlike the other samples, which exhibit sharp fluctuations and localized decreases in hardness, sample 2 maintains a stable hardness level ranging from 500 to 330 HV from the surface to a depth of 100 μm. This indicates uniform saturation of the surface with active elements (nitrogen and carbon) and the formation of a stable hardened layer without a pronounced gradient. Based on the obtained data, the optimal choice is the treatment regime and electrolyte composition corresponding to sample 2. However, in order to reduce the corrosive aggressiveness of the medium and minimize potential negative effects associated with the use of ammonium chloride, it is proposed to replace the 5% ammonium chloride with an additional 5% urea. Urea is also a source of nitrogen but is less aggressive toward metal surfaces and equipment. Such an adjustment of the electrolyte composition may preserve the advantages of the sample 2 regime while providing milder treatment conditions. Next, samples 7, 8, and 9 were treated at temperatures of 650 °C, 750 °C, and 850 °C, respectively, using the electrolyte composition and processing regime presented in Table 1.

**Figure 5:**
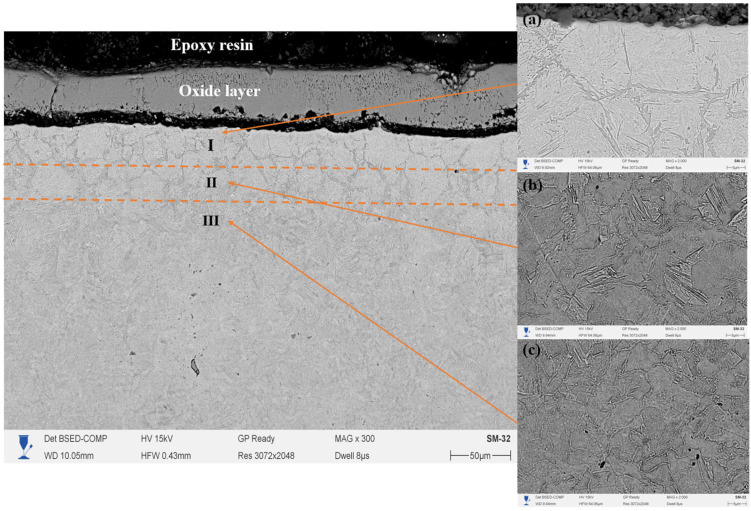
Cross-section of 20-grade steel after EPNC at 650 °C, obtained using SEM at ×300 magnification. I—nitrocarburized zone; II—transition zone; III—base structure. SEM images of the corresponding layers at ×2000 magnification: (**a**–**c**).

The cross-section of the 20-grade steel sample after EPNC conducted at a temperature of 650 °C shows a clearly defined gradient structure, characteristic of thermochemical processes involving the saturation of the surface layer with active elements. In the SEM Figure 5 (×300), three distinct structural zones can be clearly differentiated: I—the nitrocarburized zone, II—the transition zone, and III—the base structure. The upper part of the cross-section is covered with an oxide film formed as a result of the interaction between the surface and oxygen-containing components of the electrolyte under high-temperature exposure. Beneath it lies the modified layer (Zone I), characterized by high contrast and dense morphology, indicating significant changes in the phase composition and elevated concentrations of nitrogen and carbon. Upon closer examination of the microstructure (images 5a,b,c at ×2000 magnification), Zone I (5a) is characterized by the presence of elongated and oriented structural fragments, typical of iron nitrides and carbonitrides, confirming intensive saturation and redistribution of alloying elements under plasma discharge conditions. The transition zone II (5b) exhibits a gradual change in morphology, a reduction in dispersion, and the emergence of ferrite–pearlite structural fragments, indicating a gradient nature of thermal and chemical influence. In Zone III (5c), the original structure of 20-grade steel is observed, predominantly consisting of a ferrite–pearlite mixture without signs of phase transformation, confirming the limited penetration depth of active elements under the selected temperature–time regime of EPNC.

**Figure 6:**
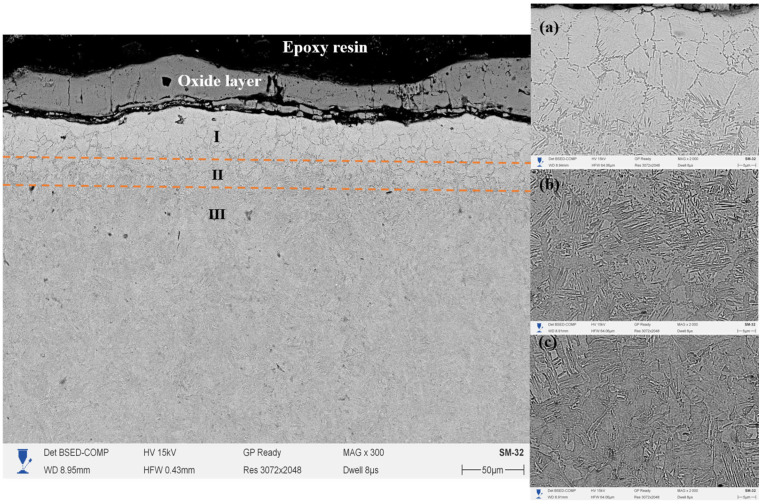
Cross-section of 20-grade steel after EPNC at 750 °C, obtained using SEM at ×300 magnification. I—nitrocarburized zone; II—transition zone; III—base structure. SEM images of the corresponding layers at ×2000 magnification: (**a**–**c**).

Figure 6 shows the cross-section of 20-grade steel after EPNC carried out at a temperature of 750 °C, obtained using a scanning electron microscope at ×300 magnification. The morphology of the treated layer is clearly divided into three main zones: I—the nitrocarburized zone, II—the transition zone, and III—the base structure. The surface region is covered with a continuous oxide film formed as a result of high-temperature interaction with electrolyte components and atmospheric oxygen, which is typical for plasma-based thermochemical processes. The microstructure of Zone I (area 6a in the SEM image at ×2000 magnification) consists of a fine-grained and dense structure with clearly defined grain boundaries. Such morphology indicates a high content of nitrogen and carbon, as well as the formation of nitride and carbonitride phases, which contribute to significant strengthening of the near-surface region. The transition zone (II) (area 6b) is characterized by the presence of acicular and lamellar precipitates uniformly distributed within the matrix, indicating ongoing phase transformations associated with gradient saturation. In this zone, there is a gradual decrease in the concentration of active elements and thermal exposure. Zone III (area 6c) represents the typical ferrite–pearlite structure of 20-grade steel without signs of thermal transformation, confirming the limited penetration of plasma heat and active elements into the material under the selected treatment regime.

**Figure 7:**
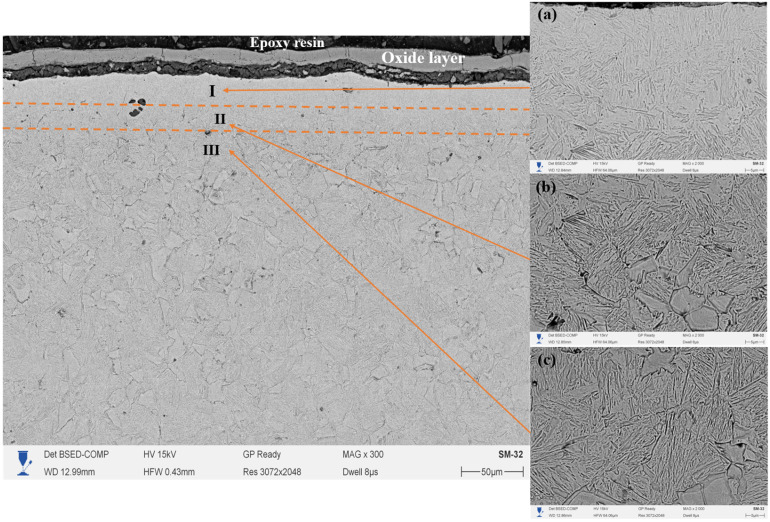
Cross-section of 20-grade steel after EPNC at 850 °C, obtained using SEM at ×300 magnification. I—nitrocarburized zone; II—transition zone; III—base structure. SEM images of the corresponding layers at ×2000 magnification: (**a**–**c**).

Figure 7 shows the cross-section of 20-grade steel after electrolytic–plasma nitrocarburizing (EPNC) performed at a temperature of 850 °C, obtained using a scanning electron microscope (SEM) at ×300 magnification. As in the previous cases (Figure 6—750 °C and Figure 5—650 °C), the microstructure is clearly divided into three zones: I—nitrocarburized zone, II—transition zone, and III—base structure. The surface is covered with a continuous oxide film formed as a result of intense interaction with the electrolyte and high-temperature exposure. The microstructure of Zone I (area 7a, ×2000 magnification) reveals a dense acicular structure typical of nitrogen-containing phases, such as nitrides and carbonitrides, formed as a result of high-temperature saturation. Zone II (7b) is characterized by a pronounced gradient morphology with regions of spheroidized ferrite and fragments of pearlite, illustrating the transition from the modified structure to the base material. Zone III (7c) retains the classical ferrite–pearlite structure typical of 20-grade steel, with large grains of pearlite and ferrite that have not undergone significant structural transformation.

A comparison of the three temperature regimes (650 °C, 750 °C, and 850 °C) allows for conclusions to be drawn regarding the influence of EPNC temperature on the morphology and depth of the modified layer. At 650 °C (Figure 5), a thin hardened layer (~40–50 μm) is formed with a well-defined transition zone and minimal phase transformations, indicating moderate diffusion and a low degree of thermal influence. At 750 °C (Figure 6), an expansion of the nitrocarburized zone is observed, along with an increase in the amount of fine-dispersed phases and a more pronounced structural gradient, confirming enhanced nitriding efficiency and activation of surface processes. With a further increase in temperature to 850 °C (Figure 7), phase transformations intensify: the thickness of Zone I increases significantly, and elongated acicular and lamellar structures characteristic of iron nitrides are formed. However, signs of overheating are observed, including local softening of the transition zone, intensive formation of an oxide film, and a possible increase in the brittleness of the hardened layer. From the standpoint of achieving a balance between saturation efficiency, thermal stability, and microstructural homogeneity, a temperature of 750 °C is optimal. It ensures stable formation of the nitrocarburized layer without the signs of overheating observed at 850 °C, while providing more pronounced modification than at 650 °C.

**Figure 8:**
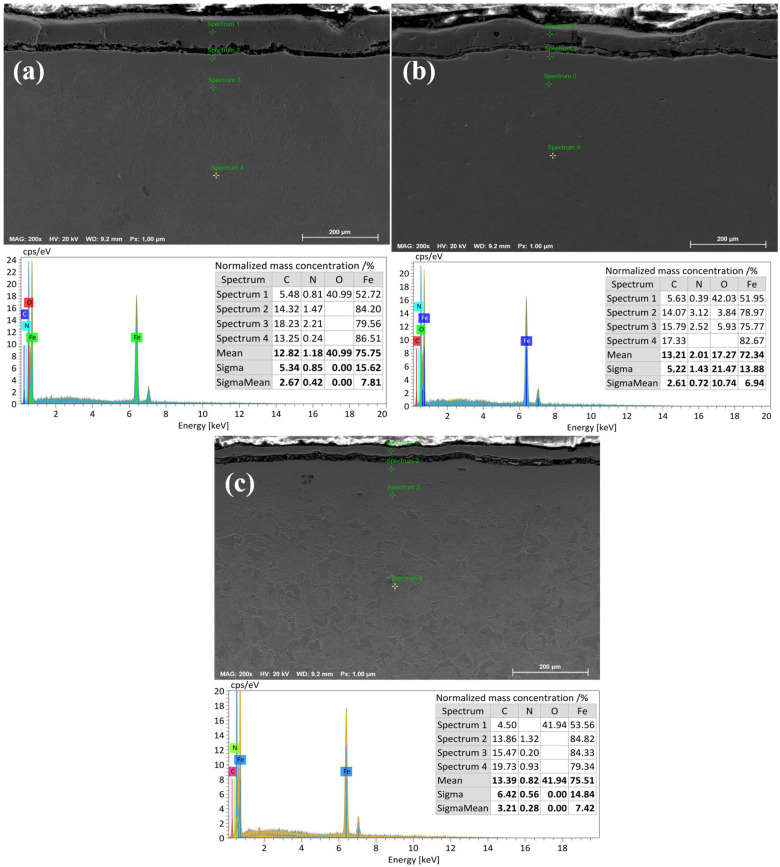
Energy-dispersive X-ray spectroscopy (EDX) of 20-grade steel after EPNC at temperatures of (**a**) 650 °C, (**b**) 750 °C, and (**c**) 850 °C.

Figure 8a presents energy-dispersive spectroscopy (EDS) data for the 20-grade steel sample after electrolytic–plasma nitrocarburizing at a temperature of 650 °C. Measurements were taken at four points across the cross-section from the surface to the substrate. The highest oxygen (O) content is observed in spectrum 1, which is located closest to the surface, due to the formation of an oxide layer. The carbon (C) concentration increases with depth, reaching its maximum in spectra 2 and 3, and then slightly decreases in spectrum 4. The nitrogen (N) concentration remains moderately low at all points, which may indicate limited nitrogen diffusion at this temperature. The average contents of C and N are 12.82% and 1.18%, respectively, while the Fe content is 75.75%.

Figure 8b illustrates the results of EDS analysis after EPNC at a temperature of 750 °C. Compared to the previous case, a more uniform distribution of carbon is observed, along with a significant increase in nitrogen concentration, particularly in spectra 2 and 3, where it reaches 3.12% and 2.52%, respectively. This indicates an increased efficiency of diffusion saturation of the surface layers with nitrogen at 750 °C. Oxygen, as before, predominates near the surface (spectrum 1—42.03%). Average elemental contents are as follows: C—13.21%, N—2.01%, O—17.27%, Fe—72.34%.

Figure 8c shows the microstructural and elemental composition of 20-grade steel after treatment at 850 °C. At this level of thermochemical activity, pronounced carbon saturation is observed, especially in spectra 3 and 4 (up to 19.73%), indicating intensified carburizing processes. However, the nitrogen content remains relatively low (averaging 0.82%), likely due to phase redistribution or partial nitrogen displacement at high temperatures. Oxygen is predominantly concentrated in the near-surface zone (spectrum 1—41.94%), forming a stable oxide layer. Average elemental contents are as follows: C—13.39%, N—0.82%, O—41.94%, Fe—75.51%.

To gain a more detailed understanding of the distribution mechanism of alloying elements across the depth of the hardened layer, in addition to point analysis, energy-dispersive spectroscopy (EDS) in line-scan mode was performed on the cross-sections of the samples after EPNC at temperatures of 650 °C, 750 °C, and 850 °C. Line-scan analysis enables the tracking of the continuous variation in the concentration of elements (Fe, C, N, O) from the surface through the oxide and nitrocarburized layers toward the base material structure.

**Figure 9:**
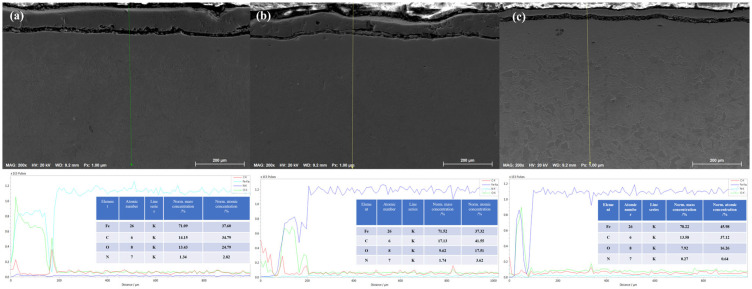
Line-scan energy-dispersive X-ray spectroscopy (EDX) of 20-grade steel after EPNC at temperatures of (**a**) 650 °C; (**b**) 750 °C; (**c**) 850 °C.

The depth distribution of elements after EPNC at 650 °C (Figure 9a) reveals a distinct near-surface oxygen (O) peak, corresponding to a thin oxide layer. The carbon (C) concentration increases with depth, reaching a maximum at the boundary of the transition zone and then stabilizing. Nitrogen (N) is detected in small amounts, and its profile indicates limited penetration, which is consistent with its low thermal activity at this temperature. The iron (Fe) content gradually increases with increasing distance from the surface. The overall pattern confirms the results of the point analysis: a thin hardened layer with a limited nitriding zone.

At a temperature of 750 °C (Figure 9b), a more uniform distribution of carbon with depth is observed, along with a significant expansion of the nitrogen penetration zone. The nitrogen profile shows a moderate concentration in the surface region and a gradual decrease toward the substrate, indicating deep diffusion and high nitriding efficiency. The oxygen concentration is still present near the surface but is lower compared to the 650 °C regime. Iron exhibits the opposite trend, gradually recovering from the oxidized layer toward the base metal. These results are consistent with the point analysis data and confirm that an optimal compositional gradient of the hardened layer is formed at 750 °C.

The line profile of the sample treated at 850 °C (Figure 9c) shows an intense oxygen peak near the surface, indicating deep oxidation and the possible formation of unstable oxides. Carbon exhibits a high and sharply defined profile in the upper region, confirming active carburization. However, the nitrogen signal remains low throughout the entire thickness of the layer, which is consistent with the effect of desorption or nitrogen displacement under overheating conditions. Iron shows a typical upward trend from the surface toward the interior. Overall, the results of the line-scan analysis confirm the point EDS data: at 850 °C, intensive carburization and oxidation occur, but nitriding remains limited.

Comparison of the three EPNC temperature regimes (650 °C, 750 °C, and 850 °C) demonstrates distinct differences in the nature of diffusion saturation of 20-grade steel. At 650 °C, limited penetration of both nitrogen and carbon is observed, attributed to the low kinetics of thermodiffusion. At 750 °C, an optimal balance is achieved: the highest nitrogen concentration (2.01%) and uniform carbon distribution (13.21%), indicating the most effective regime for nitrocarburizing. At 850 °C, despite the high carbon content (13.39%) and deep carburization, the nitrogen content decreases to 0.82%, which may be associated with nitrogen displacement from the lattice or its desorption. Additionally, the significant accumulation of oxygen near the surface at 850 °C may indicate intensified oxidation processes. Thus, the temperature of 750 °C appears to be the most optimal for forming a diffusion layer enriched with both nitrogen and carbon, without excessive surface oxidation, unlike the extreme regimes at 650 °C and 850 °C.

**Figure 10:**
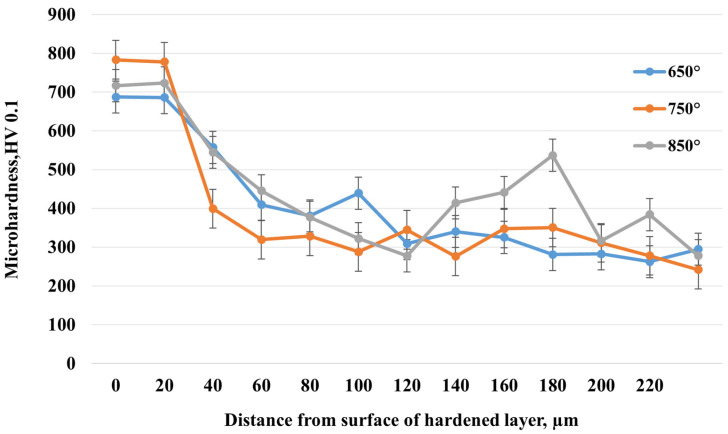
Microhardness distribution with depth after EPNC at different temperatures.

The graph (Figure 10) presents the microhardness distribution with depth of the hardened layer of 20-grade steel after electrolytic–plasma nitrocarburizing (EPNC) at three different temperature regimes: 650 °C, 750 °C, and 850 °C. All curves demonstrate behavior typical of thermochemical treatment—high hardness values in the near-surface zone followed by a decrease toward the bulk material. At 750 °C, the highest surface microhardness value of approximately 800 HV is achieved, corresponding to active saturation with both nitrogen and carbon, as confirmed by the EDS data (Figure 8). However, the hardness decreases quite sharply with depth: already at 40–60 μm, the value drops to 400–300 HV, indicating a relatively limited thickness of the diffusion layer despite its high saturation. For the 850 °C regime, the initial microhardness is slightly lower (around 730 HV), but it remains elevated to a greater depth. Values in the range of 80–200 μm remain higher compared to other regimes and also show local hardness increases (up to 550–580 HV), which may be associated with the formation of intragranular nitride or carbide phases. This indicates deeper thermodiffusion saturation but may also suggest a risk of overheating or brittleness at excessive temperatures. At 650 °C, a more moderate surface hardness value of about 700 HV is observed, with a relatively gradual decrease with depth. Despite the less pronounced maximum hardness, this regime provides the most uniform hardness distribution down to a depth of 200 μm, which may be advantageous in terms of balancing material strengthening and toughness.

**Figure 11:**
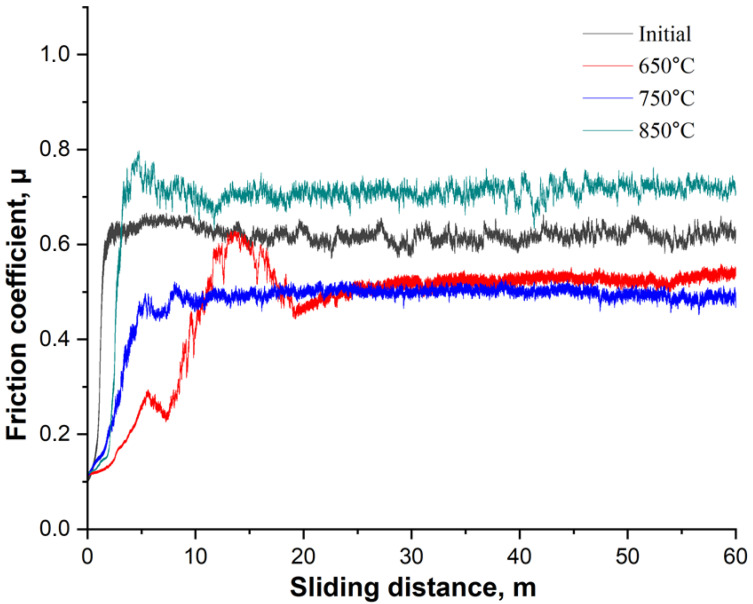
Dependence of the coefficient of friction on sliding distance for the initial sample and samples after EPNC at temperatures of 650 °C, 750 °C, and 850 °C.

Figure 11 shows the dynamics of the coefficient of friction (μ) as a function of sliding distance for 20-grade steel before and after EPNC at temperature regimes of 650 °C, 750 °C, and 850 °C. The curve for the initial (untreated) state shows an initial increase in the coefficient of friction to approximately 0.65, followed by stabilization at this level, which is characteristic of an unmodified surface without a hardened layer. The sample treated at 850 °C exhibits the highest coefficient of friction (~0.75–0.80) with significant instability and fluctuations throughout the test. This may be associated with the excessive brittleness of the surface layer, the formation of brittle oxide phases, and an unstable microrelief that promotes microdamage in the contact zone. The sample after EPNC at 650 °C exhibits an initially low coefficient of friction, stabilizing at approximately 0.55–0.60 after 10 m of sliding, which is lower compared to the initial untreated state. Nevertheless, initial fluctuations and instability are observed, indicating uneven behavior of the layer, possibly due to insufficient diffusion or thinness of the hardened zone. The most favorable results are observed for the sample subjected to EPNC at 750 °C: after a smooth initial increase, the coefficient of friction stabilizes at approximately 0.48–0.50 and remains stable throughout the test. This indicates a well-formed, homogeneous, and wear-resistant surface layer, which is also supported by the previous microhardness and compositional data (Figure 8 and Figure 10). Elevated nitrogen and carbon contents, along with a balanced microstructure, provide an optimal combination of strength, ductility, and wear resistance.

**Figure 12:**
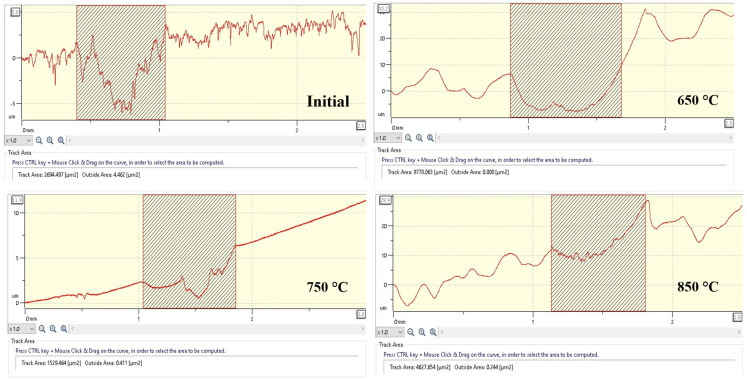
Wear track profiles and cross-sectional area (track area) for 20-grade steel samples before and after EPNC at temperatures of 650 °C, 750 °C, and 850 °C.

Based on the wear profiles, the volumetric wear index was determined (Figure 12), expressed in terms of the cross-sectional area of the wear track (µm^2^), which enables an objective comparison of the effect of EPNC temperature regimes on the tribological behavior of the material. In the initial state (Initial), 20-grade steel is characterized by high surface roughness and an unstable microrelief, with a measured wear volume of 2694.5 µm^2^, indicating poor wear resistance in the absence of surface hardening. After EPNC at 650 °C, the highest wear volume of 9778.1 µm^2^ is observed, despite a reduction in the coefficient of friction compared to the untreated state. This is explained by the formation of a thin surface layer (~40–50 µm) with limited diffusion of nitrogen and carbon, as confirmed by microstructural and spectral analyses. When the depth of surface modification is insufficient, the surface layer rapidly wears out under contact loading. The sample treated at 850 °C exhibits a moderate wear volume of 4627.7 µm^2^. Despite the deeper saturation and higher microhardness observed at this temperature, possible brittle structural degradation due to overheating and surface oxidation may have led to unstable friction behavior (Figure 11) and microcrack formation, which affected the wear surface characteristics. The minimum wear volume was recorded for the sample subjected to EPNC at 750 °C—1529.5 µm^2^, indicating the most favorable combination of properties in the hardened layer. At this temperature, optimal nitrogen and carbon content is achieved, a uniform gradient structure is formed, and high microhardness along with stable friction (~0.48) is ensured.

The specific volumetric wear was calculated using the classical tribological formula:

$$\mu =\frac{V}{F\times S}$$

where:

μ—specific volumetric wear, mm^3^/(N·m);

V—volume of worn material, mm^3^;

F—applied load, N;

S—total sliding distance, m.

To determine the wear volume, the cross-sectional area values of the worn groove obtained from profilometric data were used, with a fixed wear track length of 5 mm. The calculations were carried out under a load of 6 N and a sliding distance of 60 m. The obtained values of specific volumetric wear were as follows: without treatment (Initial)—0.000037 mm^3^/(N·m); after EPNC at 650 °C—0.000136 mm^3^/(N·m); at 750 °C—0.000021 mm^3^/(N·m); and at 850 °C—0.000064 mm^3^/(N·m). The minimum specific wear value was obtained at an EPNC temperature of 750 °C, confirming the optimality of this treatment regime in terms of improving the wear resistance of the material.

**Figure 13:**
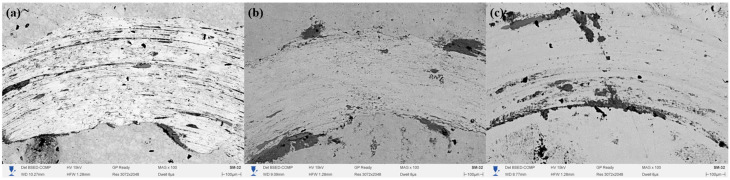
SEM micrographs of the worn surface of 20 steel after EPNC at different temperatures: (**a**) 650 °C, (**b**) 750 °C, (**c**) 850 °C.

Figure 13 presents SEM micrographs of the worn surfaces of 20 steel after EPNC at temperatures of 650 °C, 750 °C, and 850 °C. At 650 °C (Figure 13a), pronounced groove formation, localized zones of plastic deformation, and chipping are observed, which corresponds to the maximum wear volume—9778.1 µm^2^. This is due to the formation of a thin surface layer with limited diffusion, incapable of effectively resisting contact loads. At 850 °C (Figure 13c), the surface morphology is characterized by the presence of microcracks, brittle fractures, and oxidation zones, which is attributed to overheating and reduced ductility of the hardened layer. The wear volume in this case amounts to 4627.7 µm^2^. At the same time, the surface obtained after EPNC at 750 °C (Figure 13b) exhibits the lowest wear level (1529.5 µm^2^), a uniform morphology without significant defects, and stable friction behavior (coefficient ~0.48). This is associated with the formation of a gradient structure with optimal nitrogen and carbon distribution and high microhardness.

## 4. Conclusions

The conducted comprehensive study demonstrated that the electrolytic–plasma nitrocarburizing (EPNC) of 20-grade steel is an effective method of surface hardening, where the properties of the treated layer depend on the electrolyte composition and processing temperature regime. In the first stage, the nature of the plasma discharge in various electrolytes was analyzed using optical spectroscopy and high-speed video recording. Spectral analysis allowed for the identification of active plasma components, including hydrogen lines, carbon–nitrogen radicals (CN), atomic oxygen, and iron, as well as the estimation of electron density. The high-speed camera enabled the visualization of the transition from arc to glow discharge mode, which significantly affects the uniformity and depth of the hardened layer. It was found that the addition of glycerol to the electrolyte contributes to increased emission intensity and a more stable microplasma.

At the next stage, the influence of temperature (650 °C, 750 °C, 850 °C) on the microstructure, phase state, and chemical composition of the modified layer was investigated. SEM and EDS methods demonstrated that the best saturation with active elements (nitrogen and carbon) occurs at 750 °C, resulting in the formation of a uniform gradient structure with a distinctly defined nitrocarburized zone. Microhardness analysis confirmed the achievement of maximum values (~800 HV) at the surface with a gradual decrease in depth, characteristic of thermochemically hardened layers. Tribological tests revealed that at 750 °C, the lowest and most stable coefficient of friction (~0.48) is achieved, which is directly related to the uniformity of the structure and optimal content of hard phases. In contrast, at 850 °C, despite the greater depth of hardening, signs of overheating, unstable friction, and oxidation are observed, whereas at 650 °C, the layer is less saturated and exhibits reduced wear resistance.

Thus, a temperature of 750 °C combined with an optimized electrolyte provides the best combination of strength, wear resistance, and stability of the surface layer, making this regime the most promising for industrial applications of EPNC aimed at enhancing the operational reliability of 20-grade steel products.

## Figures and Tables

**Table 1:** Treatment parameters and electrolyte compositions used.

Samples	Electrolyte	Treatment Mode and Duration	Treatment Temperature
Sample 1	10% soda ash, 15% urea, 5% ammonium chloride, remainder distilled water (Electrolyte 1)	320 V, 10 s; 180 V, 5 min	750 °C
Sample 2		320 V, 10 s; 180 V, 7 min	
Sample 3		320 V, 10 s; 180 V, 10 min	
Sample 4	10% soda ash, 15% urea, 5% ammonium chloride, 3% glycerol, remainder distilled water (Electrolyte 2)	320 V, 10 s; 180 V, 5 min	750 °C
Sample 5		320 V, 10 s; 180 V, 7 min	
Sample 6		320 V, 10 s; 180 V, 10 min	
Sample 7	10% soda ash, 20% urea, remainder distilled water	320 V, 10 s; 180 V, 7 min	650 °C
Sample 8			750 °C
Sample 9			850 °C

**Table 2:** Calculated spectrogram data.

Spectrum	ΔλF, nm	ΔλL, nm	Log_10_n_e_	Electron Density n_e_, cm^−3^
(a)	~1.20	0.475	~16.20	~1.6 × 10^16^
(b)	~1.25	0.528	~16.23	~1.7 × 10^16^
(c)	~1.30	0.492	~16.18	~1.5 × 10^16^
(d)	~1.10	0.262	~15.95	~1.0 × 10^16^

## Data Availability

The original contributions presented in the study are included in this article; further inquiries can be directed to the corresponding author.
